# The Adverse Maternal Outcomes and Risk Factors of Adolescent Pregnancy: Evidence From a Retrospective Study in Astana, Kazakhstan

**DOI:** 10.3389/ijph.2025.1608992

**Published:** 2026-01-26

**Authors:** Saule Bekenkyzy Derbisbek, Aigul Abduldayevna Abduldayeva, Nailya Delellis, Zaituna Gadilovna Khamidullina, Dina Kalen

**Affiliations:** 1 Department of Research Institute of Preventive Medicine Named Academician E. Dalenov, and Department of Obstetrics and Gynecology 1, Astana Medical University, Astana, Kazakhstan; 2 School of Health Sciences, Central Michigan University, Mount Pleasant, MI, United States

**Keywords:** adolescent pregnancy, teenage pregnancy, obstetric complications, maternal outcomes, Kazakhstan

## Abstract

**Objectives:**

Adolescent pregnancy remains a global health concern. Evidence from Central Asia is limited, where sociocultural factors and evolving healthcare systems may influence outcomes. This study compared maternal and perinatal outcomes between adolescent and adult mothers in Astana, Kazakhstan.

**Methods:**

A retrospective study was conducted among primigravid adolescents (10–19 years, n = 135) and adults (20–30 years, n = 106). Data extracted from an electronic MIS and analyzed using descriptive statistics.

**Results:**

No significant differences found between the groups in preeclampsia, PIH, or anemia rates. Adolescents had higher rates of STI (21.5% vs. 6.6%, p = 0.001) and postpartum endometritis (14.1% vs. 3.8%, p = 0.007). Adults exhibited higher rates of PROM (20.8% vs. 5.2%, p < 0.001) and fetal distress (64.3% vs. 18.5%, p < 0.001).

**Conclusion:**

Adolescent pregnancy outcomes in Kazakhstan differ from other regions, likely reflecting distinct healthcare access and sociocultural factors. Targeted adolescent-friendly reproductive health services, routine STI screening, and supportivepolicies enabling early and confidential prenatal care are warranted to improve maternal and neonatal outcomes.

## Introduction

Adolescent pregnancy, a pregnancy that occurs in a female under the age of 20 remains a significant global public health challenge [1]. Commonly, adolescence is divided into three developmental stages: an early adolescence stage between 10–14 years; a middle adolescence stage 15–17 years old, and a late adolescence of 18 years and older [2]. Although the exact age ranges may differ slightly across sources, this classification helps to distinguish the unique physical, cognitive, and social developments that occur at each stage.

Worldwide, approximately 21 million girls become pregnant annually, and about 12 million of these pregnancies result in childbirth [3]. Although global adolescent birth rates have declined over the past 2 decades, significant regional differences remain, particularly in low- and middle-income countries (LMICs), where socioeconomic barriers, limited access to contraception, and cultural norms contribute to persistently high rates [4].

Kazakhstan, a middle-income, post-Soviet Central Asian country, has a population of 20.3 million, including 3.5 million adolescents aged 10–19 years (17.2%) [5, 6]. In 2023, adolescent births numbered 12,664 (3.26% of total births), with notable geographic variation, the highest rate of adolescent deliveries occurred in rural areas approximately 2,625 births [5]. Despite a decline in national fertility rates, the persistence of adolescent pregnancies underscores ongoing social and healthcare disparities across regions.

Studies from high- or low-income countries have consistently reported that adolescent pregnancies compared with the pregnancy of adult women, associated with an increased incidence of adverse pregnancy and neonatal outcomes, such as eclampsia, puerperal endometritis, preterm delivery [7–9], low birth weight (LBW), postpartum hemorrhage [10–12] stillbirth, small for gestational age (SGA), and neonatal death [13, 14]. The World Health Organization (WHO) identifies pregnancy-related complications as one of the leading causes of mortality among adolescents aged 15–19 years [15, 16].

Adverse perinatal outcomes in adolescent pregnancies are often attributed due to physiological and psychological immaturity [17]. Moreover, sociocultural factors specific to Central Asian societies including traditional gender roles, family intergenerational communication barriers [18, 19], educational disruption, economic pressures, and limited access to prenatal care [20, 21] significantly influence outcomes of teenage pregnancy [22–25].

Despite the recognized global importance of adolescent pregnancy, there is no comprehensive research examining maternal and perinatal outcomes from Astana, the capital city of Kazakhstan, or the broader Central Asian region. The absence of context-specific data limits the ability to test whether global patterns hold true in societies with distinct cultural and healthcare characteristics. However, Astana city is an urban middle-income setting with access to tertiary healthcare, making it suitable for investigating adolescent pregnancy outcomes in a Central Asian context. Thus, generating evidence from this setting not only fills a descriptive void but also provides a critical opportunity to determine whether associations observed in high- and low-income contexts apply to a middle-income, post-Soviet society with evolving healthcare systems. This may help refine or challenge international assumptions regarding the determinants and outcomes of adolescent pregnancy.

Previous hospital-based cohort studies from Brazil [26], Turkey [27], and Thailand [28] have reported elevated risks of preterm birth, LBW, and neonatal mortality among adolescent mothers, even after adjusting for socioeconomic factors. However, these findings remain inconsistent across regions due to differences in healthcare accessibility, nutrition, and social support. Comparing outcomes from Astana with these international counterparts will help clarify whether observed risks are universal or context-dependent. For example, studies from countries with comparable socioeconomic transitions, such as Uzbekistan, Georgia, and Eastern European nations demonstrate persistent adolescent pregnancy rates despite declining fertility overall [29]. Therefore, positioning Kazakhstan within this spectrum may allow a better understanding of regional reproductive health disparities.

Thus, the current study is a retrospective hospital-based design that examines maternal and perinatal outcomes among adolescent pregnancies compared to adult pregnancies. Hence, the availability and reliability of electronic medical records in tertiary healthcare facilities in Astana will be utilized to analyze recorded maternal and perinatal outcomes of a large patient population. We aim to identify preventive strategies, guide targeted healthcare interventions, and support evidence-based policy initiatives to improve outcomes for young mothers. This research not only fills a crucial gap in the regional maternal and child health literature, but also contributes to the global understanding of adolescent pregnancy outcomes in middle-income contexts.

## Methods

### Ethical Approval

This study was conducted in accordance with the ethical standards of the 2013 Declaration of Helsinki. The protocol was approved by the Local Bioethics Committee of NCJSC Astana Medical University (No. 12, 24 September 2024).

### Study Design

The study is a retrospective hospital-based, selected based on the availability, completeness, and reliability of hospital data to ensure the inclusion of standardized and verifiable clinical information, and assessment of real-world outcomes, hence and reducing recall bias commonly observed with survey-based designs. Confidentiality of patient information was strictly observed at all stages of data collection and analysis.

### Study Population and data Extraction

**FIGURE 1:**
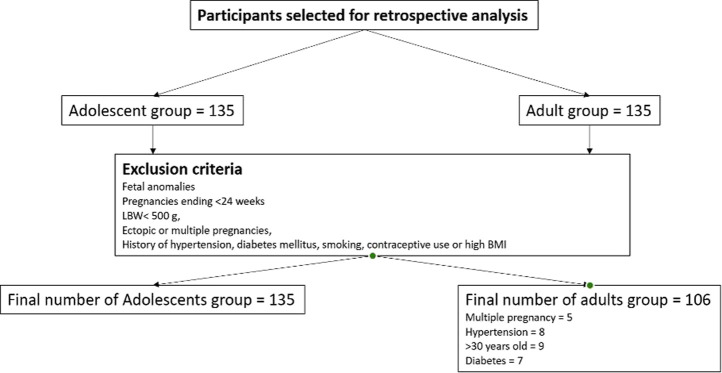
Selection process of study participant (Astana, Kazakhstan. 2025). A total of initially selected participants 270, divided equally into two groups of 135 adult participants (20–30 year old), and 135 adolescents (10–19 year old). Based on predefined criteria aimed at minimizing clinical heterogeneity and restricting the sample to low-risk singleton pregnancies, 29 participants were excluded from the adult group, leaving 106 adult mothers for analysis. The adolescent group, consisting of 135 young mothers, remained unchanged after applying the same exclusion criteria. Consequently, the final study population included a total of 241 participants: 135 adolescent and 106 adult mothers. The adverse maternal outcomes and risk factors of adolescent pregnancy: evidence from a retrospective study in Astana, Kazakhstan 2025.

The study population included all available medical records of primiparous women aged 10–19 years who delivered at the Multidisciplinary city hospital in Astana, a tertiary-level referral and teaching facility with 529 beds serving as a regional referral center and one of the largest perinatal care institutions in Central Asia, providing wide range of obstetric and neonatal services. The hospital is a JCI-accredited, and maintains a fully integrated electronic Medical Information System (MIS) that ensures standardized, routinely updated clinical data entry by obstetric and neonatal staff. Attempts to include more data from other medical institutions in Kazakhstan were unsuccessful due to denial of access to medical information and legal restrictions, limiting the sample expansion of both adolescent and adult groups. All data included in the analysis were complete. Participants categorized as adolescents (10–19 years) and adults (20–30 years) based on the WHO definition of adolescence [30]. A total of 270 participants were initially selected for this study and divided into two groups: adolescent group (n = 135) and adult group (n = 135). Note the adult age-range represents the demographic distribution with the lowest physiological risk for obstetric complications according to prior research [31, 32]. Although women aged 20–24 years may share some social and biological characteristics with older adolescents, this grouping allowed consistency with international literature and ensured adequate sample size for comparison [32]. Nevertheless, the adolescent group was divided into two subgroups: younger adolescents (10–16 years) and older adolescents (17–19 years); the adult group was divided into younger adults (20–24 years) and older adults (25–30 years). Exclusion criteria: The study focused on low risk singleton pregnancies, and to reduce clinical heterogeneity, women who were carriers of fetal anomalies, pregnancies ending before 24 weeks of gestation and/or with a birth weight <500 g, ectopic or multiple pregnancies, age over 30 years were excluded. Similarly, those who had pregestational hypertension, diabetes mellitus, smoking, history of hormonal-based contraceptive use or high BMI before pregnancy were also excluded from this study. Therefore, of the original 135 adult participants, 29 were excluded according to the predefined criteria, resulting in a final sample of 106 adult mothers. The adolescent group of 135 young mothers remained unchanged after applying the exclusion criteria. Therefore, the final study population comprised 241 participants: 135 adolescent mothers (10–19 years) and 106 adult mothers (20–30 years). The overall study selection is shown in the flow diagram (Figure 1).

### Data Collection

Data extracted by trained clinical researchers with medical backgrounds who received instruction on standardized data entry and variable coding. To ensure that the data were collected correctly, all information was reviewed and verified each day after data collection by a senior obstetrician to minimize errors and ensure data accuracy. Data entry was performed in Microsoft Office Excel. Demographic characteristics, pregnancy-related data, maternal outcomes, and neonatal outcomes were obtained from the hospital’s MIS. In cases of missing or ambiguous entries, data were crosschecked with paper-based charts to ensure completeness. Maternal age was defined as age at delivery. Gestational weeks were calculated based on the last menstrual period (LMP) or adjusted by first-trimester ultrasound when discrepancies exceeded 7 days. BMI was calculated as weight (kg) divided by height squared (m^2^) and classified according to WHO criteria. Neonatal follow-up was limited to in-hospital observation until discharge; no post-discharge or 28-day follow-up data were available, as these were reported within the hospital’s MIS. Neonatal deaths occurring during hospitalization were included. Delivery mode (vaginal or cesarean section) and fetal presentation during vaginal delivery (cephalic, breech, or other) were recorded. Preterm birth was defined as delivery before 37 weeks and further categorized into moderate-late (32–36 + 6 weeks), early (28–31 + 6 weeks), and extremely preterm (<28 weeks).

### Outcome Definitions

All outcomes were defined according to standardized clinical criteria to ensure consistency and reproducibility: Cesarean Section: Both emergency and planned cesarean deliveries, categorized according to the clinical indication and timing of the decision [33]. Anemia: Defined as hemoglobin concentration below 110 g/L, consistent with World Health Organization criteria for pregnant women [34]. Preeclampsia: Hypertension occurring after 20 weeks of gestation (systolic blood pressure ≥140 mmHg or diastolic blood pressure ≥90 mmHg) accompanied by proteinuria (≥300 mg/24 h) or evidence of end-organ dysfunction including liver dysfunction, renal insufficiency, neurological complications, or hematological abnormalities [35]. HELLP Syndrome: A severe form of preeclampsia characterized by hemolysis, elevated liver enzymes, and low platelet count (thrombocytopenia) [36]. Premature Rupture of Membranes: Spontaneous rupture of fetal membranes before the onset of labor [37]. Chorioamnionitis: Clinical diagnosis based on maternal fever, uterine tenderness, malodorous amniotic fluid, and maternal or fetal tachycardia [38]. Postpartum Puerperal Endometritis: Clinical diagnosis of uterine infection occurring within the postpartum period, characterized by fever, uterine tenderness, and purulent lochia [39]. Preterm Birth: Delivery occurring before 37 completed weeks of gestation, calculated from the last menstrualperiod or early ultrasound dating [40]. Low Apgar Score: Apgar score less than 7 at either 1 or 5 min after birth, indicating potential neonatal compromise [41].

### Statistical Analysis

Statistical analyses were performed using IBM SPSS Statistics version (26.0). The normality of continuous variables was assessed using the Kolmogorov-Smirnov test. Normally distributed continuous variables are presented as mean ± standard deviation (M±SD), while non-normally distributed variables are reported as median with interquartile range [Me (Q1-Q3)]. Categorical variables are described using frequencies and percentages. For continuous variables, the Mann- Whitney U test was used for non-parametric data and the independent t-test for parametric data.Categorical variables were analyzed using Pearson’s chi-square test. Multivariable logistic regression analysis was performed to adjust for potential confounding factors including maternal education, marital status and delivery outcomes. Variables with p-values <0.05 in univariable analysis were considered for inclusion in the multivariable model. Results are presented as risk ratios (RR) or adjusted risk ratios (aRR) with 95% confidence intervals (CI). Statistical significance was defined as p < 0.05 for all analyses. All tests were two-tailed, and no adjustments were made for multiple comparisons in the study.

### Sample Size and Power Calculation

Sample size calculations were conducted *a priori* using G*Power software (version 3.1.9.7, Heinrich- Heine-Universität Düsseldorf, Germany) [42]. The primary outcomes in this study were binary (e.g., infection, delivery mode, complications) and analyzed using logistic regression, sample size estimation was instead based on detecting an odds-ratio difference between two independent proportions. To ensure methodological consistency with the logistic regression framework, the expected effect size was expressed in terms of an odds ratio rather than Cohen’s *d*. We assumed an odds ratio of 2.0 between adolescent and adult pregnancies for key maternal or perinatal outcomes, representing a moderate association that is clinically meaningful and consistent with previous epidemiological literature [43]. This approach was guided by recommendations for logistic-regression sample-size planning in observational epidemiology, emphasizing clinically interpretable effect measures (odds ratios) and sufficient events-per-variable to ensure model stability [44]. The power analysis was conducted for a two-tailed logistic model with α = 0.05, power = 0.80, allocation ratio = 0.8, and an assumed odds ratio of 2.0 between exposure (adolescent pregnancy) and adverse outcome. This produced a minimum required 210 participants (approximately 120 adolescents and 90 adults). Our final sample of 241 participants exceeded the required minimum, ensuring adequate power (>0.80) to detect moderate associations while allowing for potential missing data. This revision aligns the sample-size justification with the actual analytical framework (logistic regression) and improves the validity of the power estimation. Covariates were included in the final multivariable models regardless of statistical significance to adjust for potential confounding.

Missing data, from both EMS and charts, were evaluated for all variables and dealt with according to Harrell et al., 2021. For example, Missing data less than 5% of the observations were deleted, and in cases of missing variables exceeding 5%, multiple imputation by chained equations was performed under a missing-at-random assumption [45]. Results from imputed and non-imputed datasets were comparable.

## Results

Data were available for 2,703 primigravid women recruited from a tertiary referral hospital in Kazakhstan between 2021 and 2024. The study population comprised 135 adolescent mothers (10–19 years) and 106 adult mothers (20–30 years).

Significant demographic differences were observed between groups (Table 1). The median maternal age at delivery was 17 years (IQR: 16–17) for adolescents versus 26 years (IQR: 23–29) for adults (p < 0.001). Adolescents reported earlier sexual initiation at 16 years (IQR: 15–16) compared to 22 years (IQR: 19–25) in adults (p < 0.001). Marital status differed substantially between groups, with 54 (40%) adolescents being unmarried compared to only 9 (8.4%) adults (p < 0.001). Educational attainment was markedly lower among adolescents: 68 (50.4%) had only middle school education versus none in the adult group, while no adolescents had university education compared to 86 (81.1%) adults (p < 0.001). Prenatal care initiation occurred significantly later among adolescents at median 14 weeks (IQR: 11–22) versus 14 weeks (IQR: 9–12) in adults (p = 0.001), with adolescents having fewer than 8 antenatal visits during pregnancy (Table 1).

**TABLE 1:** Characteristics of the adolescent pregnancies and adult groups. The adverse maternal outcomes and risk factors of adolescent pregnancy: evidence from a retrospective study in Astana, Kazakhstan 2025.

​	Adolescent group (n = 135)	Adult group (n 106)	P -value
Maternal age at delivery, range (years)	17 (16–17)	26 (23–29)	<0.001
Age of sexual initiation	16 (15–16)	22 (19–25)	<0.001
Marital status	​	​	<0.001
Married	81 (60%)	97 (91.5%)	​
Unmarried	54 (40%)	9 (8.4%)	​
Level of education	​	​	<0.001
Middle school	68 (50.4%)	0 (0%)	​
College	67 (49.6%)	20 (18.9%)	​
University	0 (0%)	86 (81.1%)	​
Initial prenatal care	​	​	<0.001
Weeks	14 (11–22)	14 (9–12)	​

Multivariable analysis revealed that compared to adult mothers, adolescent mothers had significantly reduced odds of being married (OR = 0.124, 95% CI: 0.056–0.275, p < 0.001) and achieving higher educational levels (OR = 0.247, 95% CI: 0.126–0.485, p < 0.001) (Table 2).

**TABLE 2:** Odds ratio of various risk variables for adolescent mothers’ reproductive outcomes. The adverse maternal outcomes and risk factors of adolescent pregnancy: evidence from a retrospective study in Astana, Kazakhstan 2025.

Variable	OR^a^	95% CI^b^	P -value
Marital status Married and unmarried	0.124	0.056–0.275	<0.001
Level of education	0.247	0.126–0.485	<0.001

^a^ OR, odd ratio.

^b^ CI , confidence interval.

Mode of delivery differed significantly between groups (Table 3). Vaginal delivery was more frequent among adolescents (100, 74.0%) compared to adults (44, 41.5%) (p < 0.001). Epidural anesthesia was used higher in adolescents (16, 11.9%) versus adults (4, 3.8%) (p = 0.024). Cesarean delivery rates were significantly lower among adolescents: planned cesarean sections were completely absent in the adolescent group (0%) versus 9 cases (8.4%) in adults (p < 0.001), while emergency cesarean sections also occurred less frequently (19, 14.1% vs. 23, 21.7%, p < 0.001). Labor induction was required less frequently in adolescents (13, 9.6%) compared to adults (12, 11.2%) (p < 0.001) (Table 3).

**TABLE 3:** Comparison of delivery model. The adverse maternal outcomes and risk factors of adolescent pregnancy: evidence from a retrospective study in Astana, Kazakhstan 2025.

Mode of delivery	Adolescent group (n = 135)	Adult group (n 106)	P -value
Vaginal	100 (74.0%)	44 (41.5%)	<0.001
Vaginal + epidural block	16 (11.9%)	4 (3.8%)	0.024
Vaginal delivery with episotomy	9 (6.7%)	11 (10.4%)	0.300
Vaginal delivery with perineal tear	16 (11.9%)	21 (19.8)	0.089
Planned cesarian section	0 (0%)	9 (8.4%)	<0.001
Emergency cesarian section	19 (14.1%)	23 (21.7%)	<0.001
Vacuum extraction (instrumental vaginal delivery)	3 (2.2%)	14 (13.2%)	<0.001
Labor induction	13 (9.6%)	12 (11.2%)	<0.001

No statistically significant differences were observed between groups for most maternal complications, including anemia (100, 74.1% vs. 72, 67.9%, p = 0.295), preeclampsia (8, 5.9% vs. 6.5.7%, p = 0.930), and pregnancy-induced hypertension (PIH) (15, 11.1% vs. 12, 11.3%, p = 0.959). However, adolescents demonstrated significantly higher rates of sexually transmitted infections (STI) (29, 21.5% vs. 7, 6.6%, aRR = 0.258, 95% CI: 0.108–0.617, p = 0.001) and postpartum puerperal endometritis (19.14.1% vs. 4, 3.8%, aRR = 4.177, 95% CI: 1.376–12.681, p = 0.007). Conversely, adults had higher rates of premature rupture of membranes (PROM) (22, 20.8% vs. 7, 5.2%, aRR = 4.789, 95% CI: 1.959–11.708,p < 0.001) and meconium-stained amniotic fluid/fetal distress (45, 64.3% vs. 25, 18.5%, aRR = 3.246.95% CI: 1.817–5.800, p < 0.001) (Table 4).

**TABLE 4:** Risks of adverse outcomes of pregnant women. The adverse maternal outcomes and risk factors of adolescent pregnancy: evidence from a retrospective study in Astana, Kazakhstan 2025.

Variable	Adolescent group (n = 135)	Adult group (n 106)	aRR^a^ (95%CI)	P -value
Anemia	0.7 (0.423–1.3)			0.295
Yes	100 (74.1%)	72 (67.9%)	​	​
No	35 (25.9%)	34 (32.1%)	​	​
Eye diseases	​	​	34 (7.998–146.2)	<0.001
Yes	2 (1.5%)	36 (34%)	​	​
No	133 (98.5%)	70 (66%)	​	​
Urinary tract infections	​	​	1.6 (0.792–3.24)	0.188
Yes	17 (12.7%)	29 (18.9%)	​	​
No	117 (87.3%)	86 (81.1%)	​	​
Gastrointestinal disorders	​	​	3.1 (0.78–12.3)	0.090
Yes	3 (2.2%)	7 (6.6%)	​	​
No	132 (97.8%)	99 (93.4%)	​	​
Thyroid diseases	​	​	2.7 (1.4–5.5)	0.004
Yes	15 (11.1%)	27 (25.5%)	​	​
No	120 (88.9%)	79 (74.5%)	​	​
HELLP syndrome	​	​	2.3 (0.66–8.13)	0.179
Yes	4 (3.0%)	7 (6.6%)	​	​
No	131 (97.0%)	99 (93.4%)	​	​
Amniotic fluid volume disorders	​	​	1.71 (0.73–4.02)	0.125
Yes	8 (6%)	11 (10.4%)	​	​
No	128 (94)	95 (89.6%)	​	​
Pregnancy-induced hypertension	​	​	1.021 (0.46–2.29)	0.959
Yes	15 (11.1%)	12 (11.3%)	​	​
No	120 (88.9%)	94 (88.7%)	​	​
Preeclampsia	​	​	0.95 (0.32–2.83)	0.930
Yes	8 (5.9%)	6 (5.7%)	​	​
No	127 (94.1%)	100 (94.3%)	​	​
Premature rupture of the membranes	​	​	4.79 (1.96–11.71)	<0.001
Yes	7 (5.2%)	22 (20.8%)	​	​
No	128 (94.8%)	84 (79.6%)	​	​
Meconium-stained	​	​	3.246 (1.82–5.8)	<0.001
Amniotic	​	​	​	​
fluid\FETAL	​	​	​	​
Distress	​	​	​	​
Yes	25 (18.5%)	45 (64.3%)	​	​
No	110 (81.5%)	61 (35.7%)	​	​
Chorioamnionitis	​	​	1.097 (0.36–3.37)	0.871
Yes	7 (5.2%)	6 (5.7%)	​	​
No	128 (94.8%)	100 (94.3%)	​	​
STI	​	​	0.26 (0.11–0.61)	0.001
Yes	29 (21.5%)	7 (6.6%)	​	​
No	106 (78.5%)	99 (93.4%)	​	​
Postpartum puerperal endometritis	​	​	4.18 (1.38–12.7)	0.007
Yes	19 (14.1%)	4 (3.8%)	​	​
No	116 (85.9%)	102 (96.2%)	​	​
Vacuum aspiration in postpartum period	​	​	2.75 (0.74–10.24)	0.118
Yes	10 (7.4%)	3 (2.8%)	​	​
No	125 (92.6%)	103 (97.2%)	​	​
Treatment with antibiotic	​	​	0.25 (0.11–0.57)	0.233
Yes	37 (72.6%)	22 (79.2%)	​	​
No	98 (27.4%)	84 (20.8%)	​	​

^a^ aRR , adjusted risk ratios.

Significant differences were observed in neonatal outcomes (Table 5). Median birth weight was lower among infants born to adolescent mothers (3054g, IQR: 2,750–3,340) compared to adult mothers (3382g, IQR: 2,922–3,680) (p = 0.001). Apgar scores showed subtle, but statistically significant differences, with both groups having median scores of 8, though the distribution differed (p < 0.001).

**TABLE 5:** Fetal outcome in term of gestational age, weight and Apgar score. The adverse maternal outcomes and risk factors of adolescent pregnancy: evidence from a retrospective study in Astana, Kazakhstan 2025.

Variable	Adolescent group (=135)	Adult group (n = 106)	P -value
Birth weight	3,054 (2,750–3,340)	3,382 (2,922–3,680)	0.001
Position variety	0	4 (3.8%)	0.001
Apgar score	8 (8–8)	8 (8–8)	<0.001
Gestational age	​	​	0.303
<37 weeks	121 (89.6%)	99 (93.4%)	​
>37 weeks	14 (10.3%)	7 (6.6%)	​

However, since both groups had median scores of eight, indicating generally good neonatal condition, this observed difference might not be clinically meaningful, and likely reflects minor distributional differences rather than a true disparity in neonatal wellbeing. Gestational age distribution was similar between groups, with preterm delivery (<37 weeks) occurring in 14 (10.3% adolescents versus 7 (6.6%) adults (p = 0.303).

## Discussion

This retrospective cohort study examining pregnancy outcomes among 241 primigravid women in Kazakhstan identified significant differences between adolescent and adult mothers. Key findings include higher rates of vaginal delivery and epidural anesthesia use among adolescents, against increased risks of sexually transmitted infections and postpartum endometritis. Conversely, adult mothers experienced more premature rupture of membranes and fetal distress, while their infants had higher birth weights.

Our findings regarding the demographic profile of adolescent mothers align with global patterns documented in the literature. The higher rates of unmarried status (40% vs. 8.4%) and lower educational attainment among adolescents reflect broader socioeconomic vulnerabilities associated with teenage pregnancy [46–49]. This was not unexpected, as age increases, individuals are more likely to have attained higher education levels and to be married. However, these social determinants have profound implications for maternal and child health outcomes and highlight the need for comprehensive public health interventions targeting adolescent reproductive health.

Comparable hospital-based cohort studies from Kyrgyzstan, Uzbekistan, and Georgia have reported similar trends, with adolescent mothers demonstrating lower education levels, delayed prenatal care, and increased infection rates compared to adults [29, 30]. Studies from Eastern Europe (Romania, Ukraine) and Latin America (Brazil, Peru) likewise highlight how limited reproductive health education and early marriage traditions contribute to adolescent pregnancy prevalence and adverse outcomes [26–28]. By situating our findings within this broader regional context, our study contributes unique data from Central Asia, a region underrepresented in global maternal health research, and reinforces the shared structural determinants influencing adolescent reproductive outcomes.

The delayed initiation of prenatal care among adolescents, despite occurring at similar gestational ages, may reflect barriers including lack of pregnancy recognition, fear of disclosure, and limited healthcare access [18, 19]. This finding is consistent with studies from other low- and middle-income countries and underscores the importance of accessible, youth-friendly reproductive health services. While a higher rate of vaginal delivery observed among adolescents (74.0% vs. 41.5%), which is different from other reports, the finding is consistent with established physiological mechanisms. For instance, enhanced cervical and pelvic elasticity in younger mothers promoting vaginal delivery [50, 51]. However, another explanation might be because of older women, more often, require or opt for cesarean delivery, thus, the relative proportion of vaginal births appears higher among adolescents.

The lower birth weights among infants born to adolescent mothers (3054 g vs. 3382 g), while remaining within normal ranges, may reflect maternal nutritional status, continuing growth demands of adolescent mothers, or socioeconomic factors affecting prenatal care quality. Additionally, the lower fetal weights observed in our adolescent group may contribute to reduced obstructive complications during delivery [7, 52–55]. These findings have potential long-term implications for child development and underscore the importance of comprehensive prenatal care for adolescent mothers.

Beyond physiological factors, social and nutritional determinants likely play a critical role in mediating pregnancy outcomes among adolescents in Kazakhstan. Nutritional deficiencies, including iron and micronutrient insufficiency, are common among adolescents who are still completing their own growth trajectories, which may contribute to lower infant birth weights and increased infection susceptibility [56, 57]. Culturally, early marriage and societal stigma surrounding premarital pregnancy can delay disclosure and healthcare seeking, while restrictive legal frameworks limiting minors’ consent to medical care may further hinder timely prenatal visits. These cultural and systemic barriers, combined with economic dependency and limited access to youth-centered services, may explain both the delayed initiation of care and the elevated infection rates observed in this study.

The increased use of epidural anesthesia among adolescents may reflect heightened pain sensitivity related to neurodevelopmental factors or increased anxiety associated with first pregnancy at a young age [58, 59]. This finding warrants further investigation to optimize pain management strategies for adolescent mothers.

The lack of significant differences in major obstetric complications, including preeclampsia and pregnancy-induced hypertension, contrasts with several international reports that have found higher rates of hypertensive disorders among adolescent mothers [53, 60]. This inconsistency may be attributed to variations in study populations, healthcare systems, or sample size constraints. Additionally, our recent study that focused on the southern region of Kazakhstan (refer to Ayazbekov et al.), may not fully represent other regions, such as the northern or central parts of the country, where differences in healthcare accessibility, population characteristics, environmental exposures, and regional health policies could influence outcomes [61]. Cross-national variations observed in studies from Europe, North America, and other developing regions may also result from differing healthcare infrastructures, genetic backgrounds, socioeconomic contexts, and prenatal care practices that affect both the occurrence and detection of hypertensive complications during adolescent pregnancies [62].

However, the significantly higher rates of sexually transmitted infections among adolescents (21.5% vs. 6.6%) represent a critical public health concern. This finding likely reflects risky sexual behaviors, limited access to contraception, and inadequate sexually transmitted infection screening and treatment. The associated increased risk of postpartum endometritis (14.1% vs. 3.8%) highlights the cascade effect of untreated infections on maternal morbidity [49, 63, 64]. The findings underscore the urgent need for targeted public health interventions and policy reform.

Integrating routine STI screening and treatment within adolescent antenatal care protocols could reduce infection-related complications such as postpartum endometritis. Introducing comprehensive sexual and reproductive health education within secondary schools, alongside accessible community counseling, could empower adolescents with knowledge to prevent early and unintended pregnancies.

Moreover, revising Kazakhstan’s legal framework on minors’ consent to confidential reproductive health services would improve timely access to prenatal care and contraception. Strengthening these multi-sectoral policies would align Kazakhstan with WHO recommendations for adolescent health and help reduce preventable maternal and neonatal complications.

### Study Limitation

A potential limitation of our study is that the comparison between adolescents (10–19 years) and adults (20–30 years) may introduce some degree of misclassification, as younger adults (20–24 years) can share similar behavioral and socioeconomic risk factors with older adolescents. However, this classification follows WHO criteria and is consistent with most prior studies on adolescent pregnancy. A second limitation is related to the fact that the results are based on a single-center, with retrospective design: however, the finding will enrich the evidence base from Central Asia region, and are comparable with other global regions. Another limitation is the lack of propensity score methods, and since the study groups defined *a priori* and not randomly assigned, potential residual confounding cannot be ruled out. However, the main aim was descriptive rather than causal inference, and key baseline characteristics were relatively comparable between groups. Therefore, future studies with larger samples and propensity-based adjustments are warranted to further validate these findings.

### Conclusion

This study provides evidence on pregnancy outcomes among adolescent mothers in Astana, Kazakhstan, while contributing to the broader international literature on adolescent maternal health. Unlike findings from other regions, where adolescent pregnancies have been associated with higher rates of hypertensive complications and preterm birth, our results indicate lower incidences of such conditions, but elevated risks of infection and related morbidity. These differences may reflect Kazakhstan’s distinct healthcare context, characterized by high antenatal coverage but limited adolescent-specific reproductive health services. The combination of protective physiological factors, such as lower birth weight and increased pelvic elasticity, as well as social vulnerabilities, including unmarried status and delayed prenatal care, demonstrate the multifactorial nature of adolescent pregnancy outcomes. Thus, targeted and specific interventions such as the creation of dedicated prenatal units for adolescents, the revision of consent legislation to facilitate confidential access to reproductive healthcare, and the development of multidisciplinary care teams trained in adolescent health and psychosocial support are warranted. As Kazakhstan continues to strengthen its maternal and child health services, these findings provide health strategies to reduce adolescent pregnancy related issues and improve health outcomes for young mothers and their infants.
